# Decoding NY-ESO-1 TCR T cells: transcriptomic insights reveal dual mechanisms of tumor targeting in a melanoma murine xenograft model

**DOI:** 10.3389/fimmu.2024.1507218

**Published:** 2024-11-26

**Authors:** Alaa Alsalloum, Saleh Alrhmoun, Olga Perik-Zavosdkaia, Marina Fisher, Marina Volynets, Julia Lopatnikova, Roman Perik-Zavodskii, Julia Shevchenko, Julia Philippova, Olga Solovieva, Evgenii Zavjalov, Vasily Kurilin, Hiroshi Shiku, Alexander Silkov, Sergey Sennikov

**Affiliations:** ^1^ Laboratory of molecular immunology, Federal State Budgetary Scientific Institution Research Institute of Fundamental and Clinical Immunology, Novosibirsk, Russia; ^2^ Department of Natural Sciences, Novosibirsk State University, Novosibirsk, Russia; ^3^ Center for Collective Use SPF-vivarium ICG SB RAS, Ministry of Science and High Education of Russian Federation, Novosibirsk, Russia; ^4^ Department of Personalized Cancer Immunotherapy, Mie University Graduate School of Medicine, Tsu, Japan; ^5^ Department of Immunology, V. Zelman Institute for Medicine and Psychology, Novosibirsk State University, Novosibirsk, Russia

**Keywords:** NY-ESO-1, cancer-testis antigen, TCR T cells, adoptive transfer, xenograft, SK-Mel-37, mice model, transcriptomics

## Abstract

The development of T cell receptor-engineered T cells (TCR-T) targeting intracellular antigens is a promising strategy for treating solid tumors; however, the mechanisms underlying their effectiveness remain poorly understood. In this study, we employed advanced techniques to investigate the functional state of T cells engineered with retroviral vectors to express a TCR specific for the NY-ESO-1 157-165 peptide in the HLA-A*02:01 context. Flow cytometry revealed a predominance of naïve T cells. Gene expression profiling using NanoString technology revealed upregulation of genes encoding chemokine receptors *CCR2* and *CCR5*, indicating enhanced migration towards tumor sites. In the SK-Mel-37 xenograft model, these transduced T cells achieved complete tumor eradication. Furthermore, single-cell RNA sequencing (scRNA-seq) conducted 14 days post-TCR T cell infusion provided a comprehensive analysis of the *in vivo* adaptation of these cells, identifying a distinct subset of CD8+ effector T cells with an NK cell-like gene expression profile. Our findings indicate that NY-ESO-1 TCR-transduced T cells have the potential to mediate dual antitumor effects through both antigen-independent NK-like and antigen-specific CTL-like responses. This study underscores the potential of NY-ESO-1 TCR-T cells as potent tumor-eradicating agents, highlighting the importance of harnessing their versatile functional capabilities to refine and enhance therapeutic strategies.

## 1 Introduction

The advent of adoptive cell therapy (ACT), empowered by the engineering of antigen receptor genes in T cells, heralds a new era in cancer treatment. This innovative therapeutic approach, which involves the delivery of high-quality and abundant tumor-specific T cells to cancer patients, holds extraordinary promise for improving treatment outcomes, such as increased survival rates and enhanced tumor regression (1, 2). Significant advancements have been made in the field of cellular immunotherapy for hematologic malignancies. Nevertheless, substantial challenges remain in extending these successes to solid tumors, primarily due to the intricate dynamics of the tumor microenvironment (TME) and the precise identification of tumor-associated antigens (3, 4).

Currently, overexpressed testicular and differentiation antigens dominate the landscape of TCR-based adoptive cell immunotherapy. A notable example is New York esophageal squamous cell carcinoma-1 (NY-ESO-1), a cancer-testis antigen primarily found in immune-restricted sites such as the testes and placenta (5). NY-ESO-1 is prevalent in various cancers, including head and neck cancer, multiple myeloma, metastatic melanoma, and breast cancer (6–10). Remarkably, NY-ESO-1 functions as both a tumor-associated antigen and an intrinsic adjuvant, suggesting a potential role as a damage-associated molecular pattern (11). Additionally, NY-ESO-1 triggers spontaneous antibody responses in approximately 50% of patients with NY-ESO-1-expressing tumors, a response rate significantly higher than those observed for other cancer-testis (CT) antigens such as MAGE-1, MAGE-3, and SSX2 (12, 13). These characteristics position NY-ESO-1 as a potent and versatile target with minimal off-target toxicities.

Moreover, clinical trials using adoptive cell transfer with engineered T cell receptors targeting NY-ESO-1 in melanoma and synovial sarcoma have demonstrated promising objective response rates of 40-60% (14–16). Notably, these trials have not exhibited the severe off-target effects observed with other CT antigens, such as MAGE-A3, which have resulted in neurotoxicity and cardiotoxicity due to cross-reactivity with normal tissues (17, 18). Despite these promising results, the mechanisms driving the effectiveness of NY-ESO-1-based therapies remain elusive.

Single-cell experimental methodologies have become increasingly crucial for elucidating the intricacies of T cell populations. The application of single-cell RNA sequencing (scRNA-seq) has transformed cancer research, providing comprehensive insights into the phenotypes of individual immune cells and their roles in immune responses (19–21). This state-of-the-art technology has facilitated the identification of novel states of T cell differentiation, emphasizing their adaptability during immune responses. This adaptability can be attributed to the dynamic genetic programs that occur within individual cells and the diverse composition of states observed within a population (22, 23).

Extending our previous research, in which we introduced a T cell receptor (TCR) targeting the NY-ESO-1 157-165 peptide in the context of HLA-A*02:01, we observed that retroviral transduction of these TCR genes into human lymphocytes resulted in stable transgene expression and demonstrated specific cytotoxicity against NY-ESO-1 positive tumor cell lines (24). In light of these compelling findings, we investigated whether human lymphocytes armed with the NY-ESO-1-specific TCR could inhibit the growth of NY-ESO-1-expressing tumors when adoptively transferred into an NRG immunodeficient murine SK-Mel-37 xenograft model. Subsequent *in vivo* functional assessment by single-cell RNA sequencing (scRNA-seq) revealed significant changes in gene expression of T cell subsets, indicating the emergence of effector NK-like CD8+ T cells that may have killed tumors through both antigen-specific and NK-like mechanisms.

## 2 Materials and methods

### 2.1 Engineering anti-CD3 primed peripheral blood mononuclear cells using retroviral vectors expressing TCR specific for NY-ESO-1

The methodologies employed are fully described in a previous publication (24).

### 2.2 Evaluation of the efficiency of transduction and analysis of phenotype NY-ESO-1 TCR transduced T cell

We employed MHC-biotin tetramers specific to the studied NY-ESO-1 TCR, conjugated with streptavidin-PE to assess the transduction efficiency of PBMCs. The cells (*n* = 4) were stained with these tetramers at a 1:100 dilution and incubated for 20 min in the dark at room temperature. The MHC tetramers were provided by Prof. H. Shiku (Mie University Graduate School of Medicine, Mie, Japan). After two washes, we added streptavidin-PE (Biolegend, San Diego, CA, USA), at a dilution of 1:600, along with fluorescent antibodies specific to human antigens: anti-CD8-PeCy7 (BioLegend Cat# 344712), anti-CD3-AF700 (BioLegend Cat# 300324), anti-CD4-Bv570 (BioLegend Cat# 300534), anti-CD45RA-Bv711 (BioLegend Cat# 304138), anti-CD62L-AF488 (BioLegend Cat# 304816), anti-CD95(Fas)-PerCPP-Cy5.5 (BioLegend Cat# 305630), anti-CD69-AF647 (BioLegend Cat# 310918), anti-TIM-3-APC/Cy7 (BioLegend Cat# 345026), anti-PD-1-Bv421 (BioLegend Cat# 329920), and Zombie Aqua vital dye (Biolegend, Cat#423101, San Diego, CA, USA) to evaluate the culture’s viability. Staining was conducted for 20 min at room temperature in the dark. The stained cells were then washed with PBS containing 0.1% NaN_3_ and subjected to analysis using an Attune NxT flow cytometer (Thermo Fisher, Waltham, MA, USA).

### 2.3 Flow cytometry single cell data analysis

We analyzed the data using an Attune NxT flow cytometer (Thermo Fisher, USA). We gated cells from all events, then singlets from the cells, live cells from the singlets, CD3-positive cells from the live cells, and MHC tetramer-positive cells from the live CD3-positive cells to identify the transduced T cells. We exported the gated data as.fcs files and converted them to.csv format using fcsparser. We then imported these.csv files into Seurat V5 for analysis (25).We applied data quality control (QC) (nCount_ADT < 1.000.000), followed by normalization using Centered Log-Ratio (CLR). We used Principal Component Analysis (PCA) for dimensionality reduction and performed batch correction and integration using the Harmony package (26).We applied Uniform Manifold Approximation and Projection (UMAP) for further dimensionality reduction using six Harmony-corrected principal components. We identified neighbors and performed clustering. We characterized and validated clusters using Feature Plot for markers CD4, CD8, CD62L, CD69, TIM-3, CD45RA, and PD-1. We then agglomerated and renamed clusters to reflect biological subpopulations. We created UMAP feature plots in Seurat and generated a stacked bar plot of the UMAP T cell clusters using ggplot2.

### 2.4 Cell sorting for NanoString analysis

To explore the gene expression profile with NanoString, we isolated NY-ESO-1-positive cells from the overall cell population following retroviral transduction. We accomplished this by adding an MHC-biotin tetramer to the cells (10 µL of MHC-biotin tetramer per 10 × 10^6^ cells), followed by a 20-min incubation in a cold Versene solution supplemented with 0.5% bovine serum albumin. After two washes, we added magnetic beads conjugated with MojoSort™ Streptavidin Nanobeads (Biolegend, USA, cat #480016) to the cells at a rate of 10 µL of beads per 10 × 10^6^ cells. We then washed and sorted the cells using a MojoSort™ Magnet (Biolegend, USA, cat #480019). The viability of the magnetically sorted cells was then assessed using a Countess 3 Automated Cell Counter (Thermo Fisher Scientific, Waltham, MA, USA) and trypan blue staining, revealing a cell viability of over 92%.

### 2.5 Total RNA extraction

We isolated total RNA from 450.000 - 550.00 cells with the Total RNA Purification Plus Kit (Norgen Biotek, Canada). We then measured the concentration and quality of the total RNA in each sample on a Nanodrop 2000 spectrophotometer (Thermo Fisher Scientific, USA) and froze the total RNA at −80°C until the NanoString analysis.

### 2.6 NanoString gene expression profiling

We performed gene expression profiling via the NanoString nCounter SPRINT Profiler using 100 ng of total RNA from each sample of non-transduced T cells and NY-ESO-1 TCR-transduced T cells, which were sorted with MHC-biotin tetramer (*n* = 4 for each group). We used the nCounter Human Immunology v2 panel to analyze the total RNA of the samples nCounter Human Immunology v2 panel consists of 579 immunity and inflammation-associated genes, 15 housekeeping genes, and eight negative and six positive controls. The samples were subjected to an overnight hybridization reaction at 65°C, where 8-14 μl of total RNA was combined with 3 μl of nCounter Reporter probes, 0-4 μl of DEPC-treated water, 11 μl of hybridization buffer and with 5 μl of nCounter capture probes (total reaction volume = 33 μl). After the hybridization of the probes to targets of interest in the samples, the number of target molecules was determined on the nCounter digital analyzer. We performed normalization and QC in nSolver 4 using added synthetic positive controls and the 15 housekeeping genes included in the panel. We then performed background thresholding on the normalized data to remove non-expressing genes. The background level was determined as the mean of the POS_E controls and the genes that did not pass the threshold in at least one sample were removed. We then log2-transformed the data and exported it to GraphPad Prism 9.4 software.

### 2.7 NanoString differential gene expression testing

We performed NanoString differential gene expression analysis using multiple T-tests (with Q < 0.001 and log2 (Fold Change > 3.0) in GraphPad Prism 9.4. The Volcano plot was created in GraphPad Prism 9.4.

### 2.8 *In vivo* efficacy

NRG immunodeficient mice of the NOD.Cg-Rag1tm1Mom Il2rgtm1Wjl/SzJ strain was utilized. The study was conducted at the Center for Genetic Resources of Laboratory Animals within the Institute of Cytology and Genetics, Siberian Branch of the Russian Academy of Sciences (RFMEFI62119X0023). Both male and female mice, aged 8 weeks, were included in the study, all with SPF (specific pathogen-free) status. These mice were housed in single-sex family groups, comprising 5 individuals per group, within individually ventilated cages (IVC) using the Opti Mice system provided by Animal Care Systems. These cages maintained controlled environmental conditions, including a temperature range of 21–24°C, relative humidity levels between 30–50%, and a lighting regimen of 12:12 light: dark cycle. The mice were fed a diet from Ssniff (Soest, Germany) and had access to reverse osmosis water enriched with mineral mixture ad libitum. For the melanoma model, 5 million SK-MEL-37 tumor cells were subcutaneously implanted near the right scapula of the experimental animals suspended in 100 µL RPMI medium. The experiments strictly adhered to humane and ethical standards outlined in the European Community directive (86/609/EEC). Mice were closely monitored every 2–3 days for changes in skin condition, motor activity, and behavior. If mice displayed signs of toxicity (e.g., curvature, hunching, reduced activity), a body weight loss exceeding 20%, or a significant increase in tumor volume, they were euthanized in accordance with ethical guidelines for animal care. Planned euthanasia was carried out using CO_2_ overdose, followed by cervical dislocation. Tumor volumes were precisely determined using caliper measurements and the formula V = a × b2 × 0.52 (where ‘a’ represents length and ‘b’ represents width). When the average tumor volume reached 100 mm^3^, the mice were randomly distributed into three groups. The control group received 8 million non-transduced T cells intravenously, the experimental group received NY-ESO-1- coding retroviral construct-transduced T cells in the same manner, and the third group remained untreated. Statistical analysis was conducted using GraphPad Prism 10.0.0 (GraphPad Software, USA). The two-way ANOVA test was used to compare between the groups (*n* = 4 for each group). Tumor volumes are presented as mean ± standard error of the mean.

SK-MEL-37 tumor cells were provided by Prof. H. Shiku (Mie University Graduate School of Medicine, Mie, Japan) (24).

### 2.9 Evaluation of NY-ESO-1 TCR expression in PBMCs of a melanoma xenograft model and isolation of peripheral blood T cells

Peripheral blood (up to 5 ml) was collected in EDTA-containing tubes from mice bearing xenografted tumors expressing NY-ESO-1. Peripheral blood mononuclear cells (PBMCs) were isolated using the standard Ficoll-Urografin density gradient method (PanEco, Moscow, Russia). The cells were stained with MHC tetramers and fluorescent antibodies specific to human CD3 (Anti-CD3-AF700, BioLegend Cat# 300324, San Diego, CA, USA) in accordance with the aforementioned protocol to confirm the maintenance of NY-ESO-1-specific TCRs *in vivo.*

Subsequently, CD3-positive magnetic sorting of mononuclear cells derived from murine peripheral blood was performed using a MojoSort™ Human CD3 Selection Kit (Biolegend, USA, cat # 480134) according to the manufacturer’s protocols.

### 2.10 Sample tag sample barcoding and cell counting for BD rhapsody single-cell analysis

After cell sorting, we incubated individual samples and control T cells with Sample Tag antibodies for 20 minutes at room temperature. After three washing cycles, cells were stained with Calcein according to the BD Rhapsody Single-Cell Analysis System User Guide Revision 5.0, counted using the Attune NxT flow cytometer, pooled together, and resuspended in a cold sample buffer to a final concentration of 10 cells/µl and a volume of 620 µl for loading onto a BD Rhapsody Cartridge. The quality of cell loading into the cartridge was assessed using the InCell Analyzer 2000 using Calcein.

### 2.11 cDNA library preparation and sequencing

We performed single-cell capture and cDNA library preparation using the BD Rhapsody Express Single-Cell Analysis System (BD Biosciences), according to the manufacturer’s instructions. Briefly, we amplified cDNA (10 cycles of PCR) using the Human Immune Response Primer Panel (BD Biosciences), containing 399 primer pairs, targeting 397 different genes. We purified the resulting PCR1 products using AMPure XP magnetic beads (Beckman Coulter, Brea, California, United States) and separated the respective mRNA and Sample Tag products on the basis of amplicon size. We further amplified the purified mRNA and Sample Tag PCR1 products (10 cycles of semi-nested PCR), and purified the resulting PCR2 products by size selection. We assessed the concentration by Qubit (High-Sensitivity dsDNA Kit; Thermo Fisher). We normalized the final products to 4.5 ng/μL for the mRNA library and 1.0 ng/μL for the Sample Tag library and performed a final round of amplification (6 cycles of PCR for the mRNA library and 8 cycles of PCR for the Sample Tag library) using indexes for Illumina sequencer to prepare the final libraries. We quantified the final libraries using Qubit fluorimeter and Agilent BioAnalyzer 2100 and pooled them (~ 94/6% mRNA/Sample Tag ratio, estimated 20000 (mRNA) and 1300 (Sample Tag) read/cell) to achieve a final concentration of 2 nM. The final pooled libraries were sequenced (150 bp paired-end, 600 million clusters) on Illumina NovaSeq 6000 Sequencing System.

### 2.12 Sequencing data processing

We processed the FASTQ files obtained from sequencing using the BD Rhapsody pipeline v1.10.1L (BD Biosciences). The pipeline removed read pairs with low quality based on their read length, mean base quality score, and highest single-nucleotide frequency, analyzed remaining high-quality R1 reads in order to identify cell label and unique molecular identifier (UMI) sequences, aligned the remaining high-quality R2 reads to the reference panel sequences (mRNA) using Bowtie2, collapsed reads with the same cell label, the same UMI sequence and the same gene into a single molecule, adjusted the obtained counts by error correction algorithms, namely, recursive substitution error correction (RSEC) and distribution-based error correction (DBEC), in order to correct for sequencing and PCR errors, estimated cell counts using the second derivative analysis to filter out noise cell labels, observed one inflection point and considered cell labels after that point to be noise labels. Then, the pipeline used molecular barcoded oligo-conjugated antibodies (single-cell multiplexing kit HS; BD Biosciences) to demultiplex the samples and filter out the multiplets. The pipeline called sequencing saturation of 98% and median distribution-based error correction metric (DBEC) = 8.6 (DBEC > 8 is considered deep sequencing for BD Rhapsody.

### 2.13 Data QC and analysis in Seurat

We analyzed gene expression in the Seurat V5 (25) of the R programming language, performed quality control, merged samples, normalized the data using the SCTransform package (27), used PCA dimensionality reduction, corrected PCs for the batch effect using the Harmony package (26), and performed Uniform Manifold Approximation and Projection (UMAP) dimensionality reduction using 30 Harmony-corrected PCs. Next, we found clusters and classified T cells using canonical markers (28) and the gene expression signature of transduced T cells obtained from NanoString data analysis, demultiplexed the clusters using the Sample Tag metadata into two biological groups - control T cells and murine peripheral blood T cells, and performed intra-cluster differential gene expression using the Wilcoxon test with biological and statistical significance criteria of log2 (fold change) > 1.0 or log2 (fold change) < -1.0 and q value < 0.001. We created the UMAP feature plots and the Dot plot in Seurat. We created the stacked bar plot of the UMAP T cell clusters via ggplot2. Statistical significance of the differences in the cluster composition was calculated by multiple T-tests (*n* = 4) with q-value < 0.05 in GraphPad Prism 9.4. The GSEA of the CD8 Effector T cell DEGs was done via GSEApy (29).

## 3 Results

**Figure 1:**
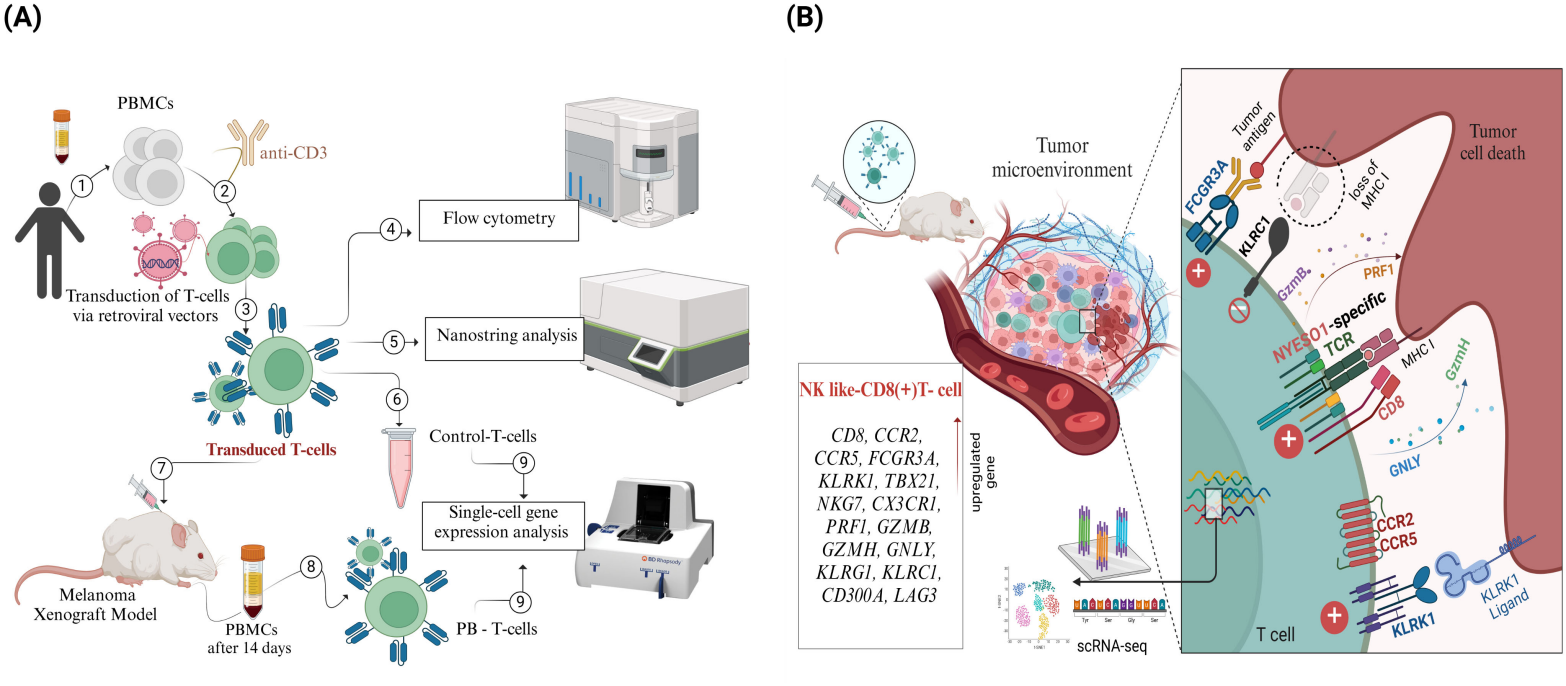
Overview of Experimental Workflow and Key Findings from the NY-ESO-1 TCR T cell Study. **(A)** Peripheral blood mononuclear cells (PBMCs) were isolated from healthy donors (1), activated using anti-CD3 antibodies (2), and transduced with retroviral vectors encoding NY-ESO-1 TCR (3). Transduction efficiency and T cell phenotyping were assessed through flow cytometry (4). Gene expression profiling was performed on transduced T cells using NanoString technology (5), and a control sample was reserved for single-cell RNA sequencing (scRNA-seq) (6). The transduced T cells were then infused into a melanoma xenograft model (7). Fourteen days post-infusion, peripheral blood T cells (PB-T cells) were isolated from treated mice (8), followed by scRNA-seq analysis of both PB-T cells and control T cells (9). **(B)** scRNA-seq analysis revealed a distinct subset of CD8+ effector T cells with an NK cell-like gene expression profile.

In our previously published study, we obtained genetically modified T cells, expressing TCR targeting NY-ESO-1 with average efficiency of 22.13 ± 7.11% (mean and standard deviation, *n* = 6), with a range from 13.40% to 33.5%, indicating the successful manufacture of NY-ESO-1-specific T cells (24). The present study employs a two-pronged approach to elucidate the phenotypic and genotypic adaptations of transduced T cells *in vivo*. This comprises a pre-infusion analysis of the immunophenotype and gene expression profile of the transduced T cells, followed by a single-cell gene expression analysis of TCR T cells from SK-Mel-37 Xenograft PBMCs on day 14 after transduced T cell infusion (**Figure 1**).

### 3.1 NY-ESO-1 TCR-transduced T cells display a predominantly naive T cell phenotype

**Figure 2:**
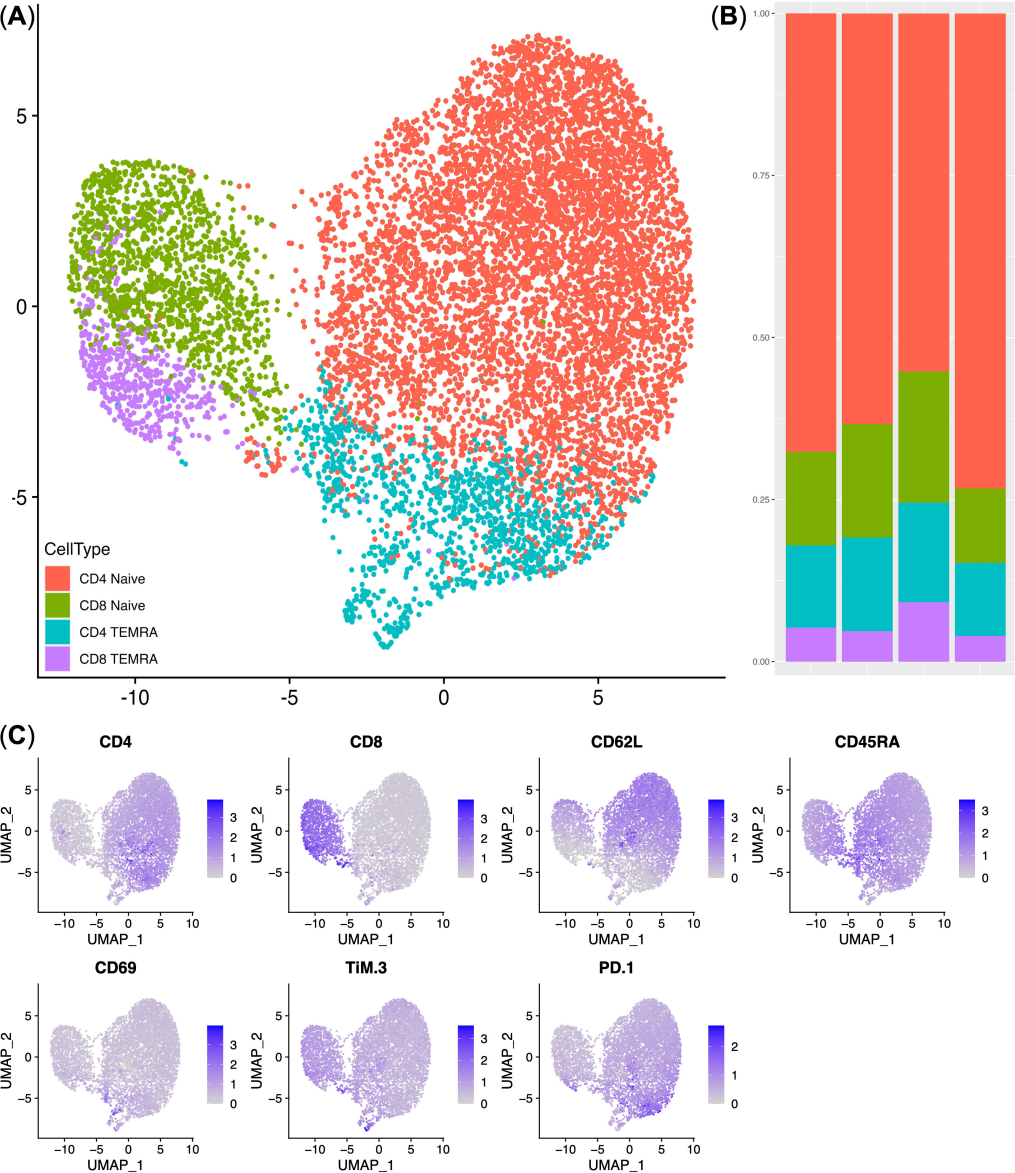
Immunophenotype analysis of NY-ESO-1 TCR T cells. **(A)** UMAP plot of the T cell clusters. **(B)** Stacked bar plot of the cell percentages in each cluster (*n* = 4). **(C)** UMAP Feature plots displaying the expression levels of cluster-defining genes. Expression levels are represented by a color scale, with grey indicating undetectable expression and deep blue representing the highest levels of expression. TEMRA — CD45RA^+^ T-effector memory cells.

Following the transduction of conditionally healthy donor cells with the NY-ESO-1 TCR construct, flow cytometry was employed to analyze the immunophenotype of the NY-ESO-1 TCR-transduced T lymphocytes. This analysis included the evaluation of T cell lineage markers (CD4 and CD8) to identify the subsets of transduced T cells, as well as markers associated with T cell memory (30, 31), including CD45RA and CD62L, and markers indicative of T cell activation and differentiation, such as CD69, PD-1, and TIM-3 (32, 33). UMAP dimensionality reduction and clustering based on gene expression profiles identified four distinct T cell subsets: CD4+ naive T cells (CD4+ CD62L+ CD45RA+), CD8+ naive T cells (CD8+ CD62L+ CD45RA+), CD4+ effector memory expressing CD45RA T cells (CD4+ TEMRA; CD4+ CD62L− CD45RA+), and CD8+ effector memory expressing CD45RA (CD8+ TEMRA; CD8+ CD62L− CD45RA+). The majority of NY-ESO-1 TCR-transduced T cells were predominantly naïve with low expression of CD69, PD-1, and TIM-3 molecules. Specifically, CD4+ naive T cells constituted 62.54% ± 9.23% of the total NY-ESO-1-specific TCR T cells, while CD8+ naive T cells comprised 12.53% ± 3.19%. Notably, a less prevalent subset of TEMRA cells exhibited moderate levels of markers CD69, PD-1, and TIM-3 (**Figure 2**).

### 3.2 NY-ESO-1 TCR-transduced T cells exhibit distinctive gene expression signature

To further validate the gene expression profile of NY-ESO-1 TCR-transduced T cells, the overall cell population was sorted using NY-ESO-1-specific MHC tetramers. Subsequently, NanoString gene expression analysis was conducted on both the NY-ESO-1-specific TCR-transduced T cells and the non-transduced, i.e., anti-CD3-stimulated PBMCs, which were not infected with a retrovirus and in which no transduction was performed. To identify the most significantly differentially expressed genes, rigorous criteria were applied. This included an extremely stringent q-value and log2 (Fold Change), enabling the selective identification of key genes, including CCR2, CCR5, ITGAM, CD96, TNFSF8, and PDGFRB (See **Supplementary Figure 1**).

### 3.3 NY-ESO-1 TCR-transduced T cells inhibit tumor growth in the SK-MEL-37 melanoma xenograft model

**Figure 3:**
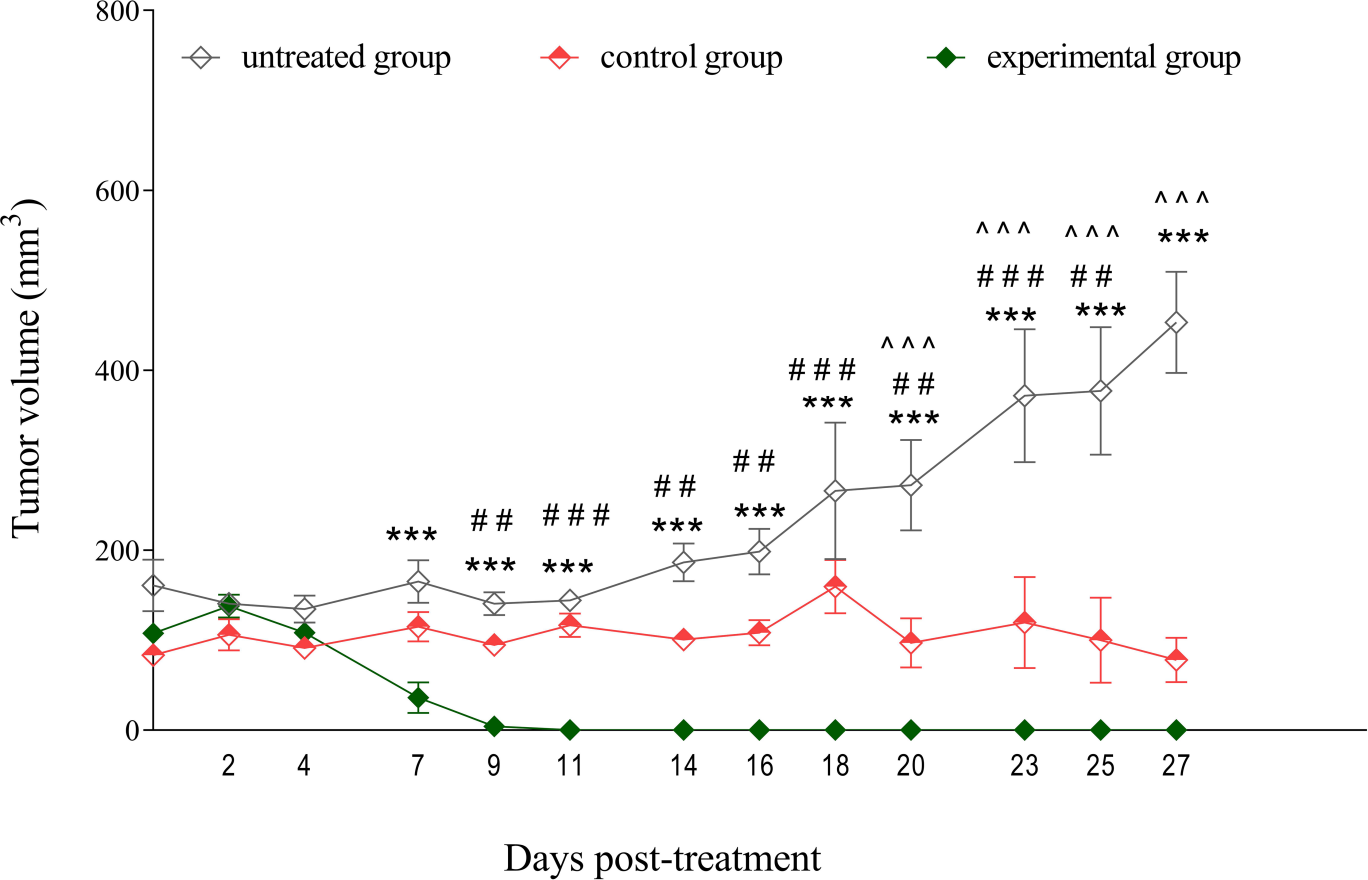
Tumor growth inhibition by NY-ESO-1 TCR-transduced T cells in a melanoma model. SK-Mel-37 tumor cells (5 million/mouse) were inoculated into NRG mice. Upon the tumors reaching sizes of 80–100 mm³, the mice were randomly allocated into three groups: control, experimental, and untreated. The control group (*n* = 4) was administered non-transduced T cells, the experimental group (*n* = 4) received NY-ESO-1 TCR-transduced T cells (8 million/mouse intravenously), and the untreated group (*n* = 4) served as a baseline for tumor growth and remained without intervention. Tumor volume was monitored over time, and the statistical significance of the differences between groups was determined using a two-way analysis of variance (ANOVA). Statistical significance between the experimental and untreated groups is denoted by asterisks (*), with corresponding p-values: ***p < 0.001. Grids (#) represent significant disparities between the experimental and control groups: ^###^ *p* < 0.001, ^##^ *p* < 0.01. Carets (^) signify significant differences between the control and untreated groups: ^^^ *p* < 0.001.

To investigate the efficacy of NY-ESO-1 TCR-transduced T cells in solid tumor model, we inoculated NRG mice with human melanoma cells SK-Mel-37 expressing NY-ESO-1. Once the average tumor volume reached 100 mm³, the mice were randomly allocated into three groups (untreated, control, experimental), with each group comprising four mice. The control group was administered non-transduced T cells, whereas the experimental group received treatment with NY-ESO-1 TCR-transduced T cells, with an approximate average transduction efficiency of 29% (**Supplementary Figure 2A**). Interestingly, these NY-ESO-1 TCR-expressing cells entirely eliminated the tumor, commencing on day 7 post-TCR T cell infusion. On the contrary, the control group (non-transduced T cells) exhibited a noticeable impediment in tumor growth progression compared to the untreated group, with this effect observable starting on day 20 after TCR T cell infusion (**Figure 3**).

### 3.4 Transduced T cells maintain NY-ESO-1 TCR expression *in vivo*

On day 14 post- transduced T cell infusion, peripheral blood samples were collected from mice with xenografted tumors. Flow cytometric analysis was conducted using NY-ESO-1-specific MHC tetramers and human anti-CD3 antibodies to quantify NY-ESO-1 TCR expression in peripheral blood T cells (PB T cells). This was compared with pre-infusion NY-ESO-1 TCR-transduced T cells, which served as controls. The average percentage of tetramer-positive cells was approximately the same as in the controls, indicating that the transduced T cells maintained their NY-ESO-1-specific TCR expression. This finding demonstrates that these T cells continued to effectively recognize the NY-ESO-1 antigen (See **Supplementary Figure 2**).

### 3.5 A subset of peripheral blood CD8 effector T cells demonstrate NK cell-like gene expression patterns

**Figure 4:**
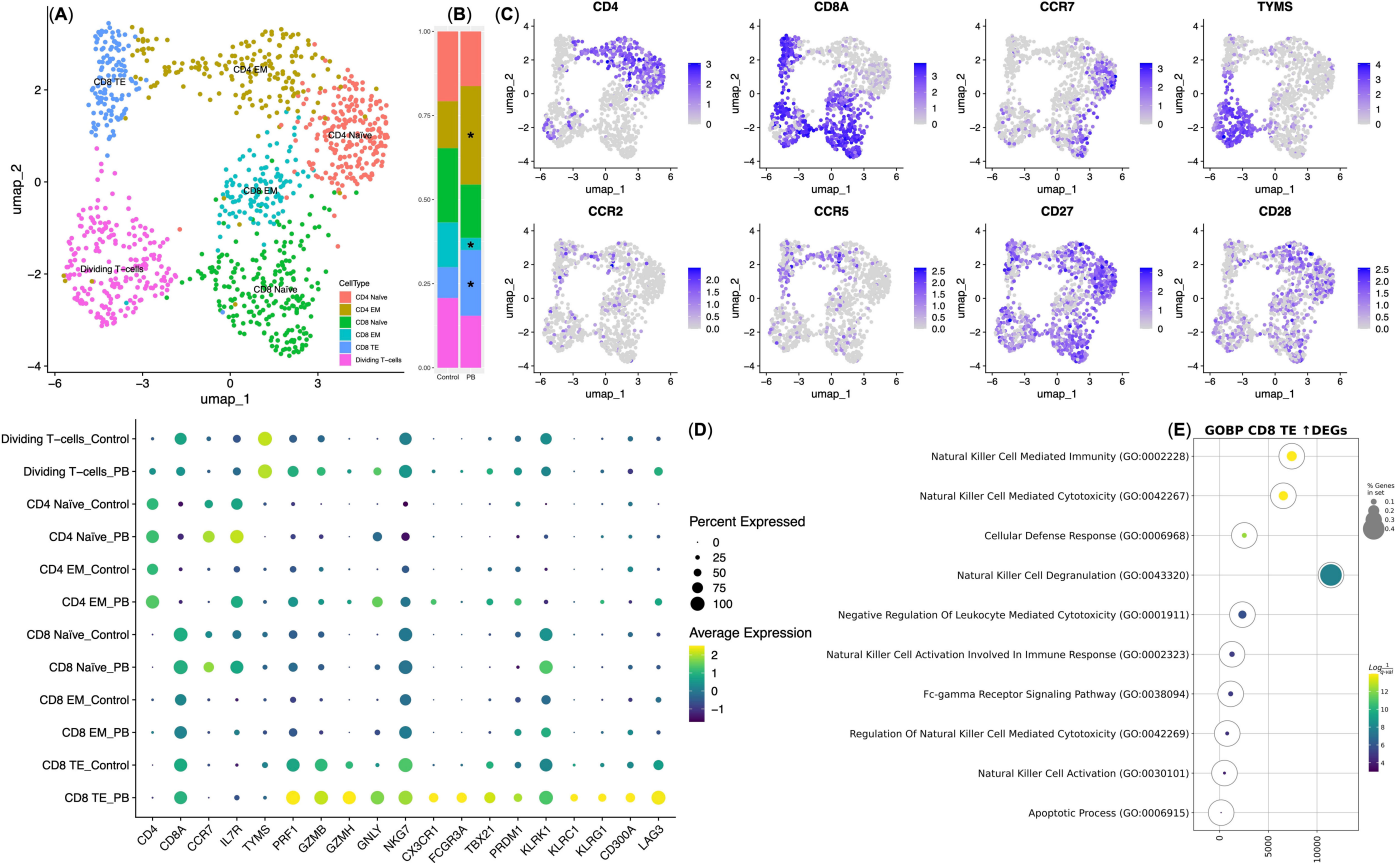
Single-cell gene expression analysis of NY-ESO-1 TCR T cells. **(A)** UMAP plot of T cell clusters. **(B)** Stacked bar plot showing cell percentages in each cluster; * indicates statistical significance in cluster composition (*q*-value *< 0.05*). Control — T cells transduced with the NY-ESO-1 TCR retroviral construct (*n* = 4); PB — T cells isolated from the peripheral blood of mice with xenografts 14 days post-infusion (*n* = 4). **(C)** UMAP feature plots of cluster-defining genes, with expression levels indicated by a color scale (grey = undetectable; deep blue = highest expression). **(D)** Dot plot of differentially expressed genes within NY-ESO-1 TCR T cell clusters. Dot size indicates the percentage of cells expressing each gene, with Z-score transformed expression levels ranging from yellow (highest expression) to deep purple (lowest expression). EM, Effector Memory T cell subset; TE, Effector T cell subset. **(E)** Bubble plot of GSEA for upregulated genes in CD8 TE PB vs. Control. The color scale represents *q*-values, with yellow indicating the lowest *q*-values (highest statistical significance) and deep purple indicating the highest *q*-values (lowest statistical significance). The size of the bubbles reflects the percentage of genes analyzed from the Gene Ontology “Biological Process” database.

To investigate the functional state of TCR T cells within the SK-Mel-37 xenograft model, we performed single-cell immune transcriptome analysis on both control T cells and PB T cells. Gene expression-based clustering identified six distinct T cell subsets: CD4 Naïve (CD4+ CCR7+ ILR7+) and CD8 Naïve (CD8+ CCR7+ ILR7+), Effector Memory CD4 EM (CD4+ CCR7− ILR7+/−) and CD8 EM (CD8+ CCR7- ILR7+/−), CD8 Effector T cells TE (CD8+ CCR7− ILR7dim), and dividing T cells (**Figures 4A, C**). Moreover, the analysis revealed that CD4 EM and CD8 TE T cell were double positive for CCR2 and CCR5, which were, in fact, NY-ESO-1 TCR-transduced T cells, based on NanoString gene expression data (See **Supplementary Figure 1**). Prior to T cell infusion, 57.4 ± 2.39% of the cells (mean ± SD, *n* = 4) exhibited gene expression profiles characteristic of naive and dividing T cells. Following infusion, we observed a significant increase in the CD4 Effector Memory (EM)T cells (29.67 ± 2.08%) and the CD8 Effector (TE) T cells (18.7 ± 2.54%), accompanied by a decrease in CD8 Effector Memory (EM) T cells (6.1 ± 1.39%) within the peripheral blood T cell population (**Figure 4B**).

We then conducted an intra-cluster differential gene expression analysis, comparing Control T cells and PB T cells, and identified significant variations primarily within the CD8 Effector (TE) T cells. This observation aligns with expectations, as CD8 TE cells are well-recognized for their predominant cytotoxic properties among TCR T cells (34). Interestingly, CD8 TE PB exhibited an upregulation in the expression of genes associated with cytotoxicity, including *PRF1*, *GZMB*, *GZMH*, and *GNLY*, along with genes encoding natural killer (NK) cell receptors, transcription factors (*NKG7, KLRK1, CX3CR1, FCGR3A, TBX21, PRDM1*), and genes encoding checkpoint inhibitory molecules (*KLRC1, KLRG1, CD300A*, and *LAG3*), in comparison to CD8 TE Control (**Figure 4D**).

Subsequently, Gene Set Enrichment Analysis (GSEA) was performed on the genes that demonstrated upregulated expression in CD8 TE PB in comparison to CD8 TE Control. Our analysis revealed an enrichment in gene signatures associated with Natural Killer cells (**Figure 4E**; **Table 1**). These specialized cells played a crucial role in the targeted and antigen-specific elimination of the SK-Mel-37 xenograft, highlighting their adept capacity to fine-tune cytotoxicity through inhibitory checkpoint molecules.

**Table 1:** GSEA of the CD8 TE PB vs Control up-regulated genes, *q*-value < 0.01.

Term	Overlap	Combined Score	Genes
Natural Killer Cell Mediated Immunity	4/22	7409.66341718403	FCGR3A, KLRK1, GZMB, NKG7
Natural Killer Cell Mediated Cytotoxicity	4/24	6544.11151961037	FCGR3A, KLRK1, GZMB, NKG7
Cellular Defense Response	4/48	2533.54010970094	CX3CR1, GNLY, PRF1, KLRG1
Natural Killer Cell Degranulation	2/5	11441.6634760018	FCGR3A, NKG7
Negative Regulation Of Leukocyte Mediated Cytotoxicity	2/14	2335.07728874903	CX3CR1, KLRC1
Natural Killer Cell Activation Involved In Immune Response	2/22	1268.17617909266	FCGR3A, NKG7
Fc-gamma Receptor Signaling Pathway	2/24	1129.82194126442	FCGR3A, FYN
Regulation Of Natural Killer Cell Mediated Cytotoxicity	2/32	772.860476757492	KLRK1, KLRC1
Natural Killer Cell Activation	2/45	493.468378653786	FCGR3A, KLRK1
Apoptotic Process	3/228	146.26499088097	PRF1, GZMB, GZMH

## 4 Discussion

Clinical trials targeting the NY-ESO-1 antigen with adoptive T cell transfer have demonstrated encouraging objective response rates (11). Nevertheless, the intricate cross-talk between T cells and tumors persists as a challenge, prompting our in-depth investigation into the microenvironment-dependent gene expression changes during T cell activation (35).

In this study, we used a two-pronged approach to investigate the adaptations of NY-ESO-1 TCR-transduced T cells in a melanoma murine xenograft model. Prior to infusion, flow cytometry showed a substantial proportion of naive T cells, particularly within the CD4+ subset (CD4+ CD62L+ CD45RA+). This naive phenotype, is typically associated with enhanced proliferative capacity and sustained anti-tumor activity (36). An increased ratio of CD4+ T has been shown to significantly improve the efficacy of adoptive T cell therapies (37–40). Additionally, the presence of a smaller subset of effector memory T cells (TEMRA; CD62L− CD45RA+), known for their ability to migrate to peripheral lymphoid tissues and deliver immediate cytotoxic responses, underscores their crucial role in early tumor eradication (30, 41).

To further investigate the functional implications of these cellular characteristics, we performed NanoString gene expression analysis, which revealed upregulation of genes related to chemokine receptors, such as *CCR2* and *CCR5*. These genes play critical roles in guiding T cell trafficking, facilitating their infiltration into tumor microenvironments (42–44). Consistent with these findings, our *in vivo* experiments demonstrated that infusion of NY-ESO-1 TCR-transduced T cells resulted in complete tumor regression starting from day 7, in contrast to non-transduced T cells.

The NanoString data also highlighted key immune functions, including the overexpression of the co-stimulatory receptor *CD96*, correlating with increased frequencies of T-bet-expressing CD8+ T cells and enhanced cytotoxic effector activity (45), and the upregulation of *ITGAM*, which is crucial for differentiating recently activated effector CD8+ T cells from memory cells (46). Additionally, the *TNFSF8* gene, a member of the TNF ligand superfamily, was expressed preferentially by activated CD4+ T cells (47). This suggests that the presence of inhibitory receptors TIM3 and PD1 on TEMRA cells is driven more by differentiation and activation rather than classical exhaustion (32).

Following infusion of NY-ESO-1 TCR-transduced T cells, single-cell transcriptomic analysis of peripheral blood T cells (PB-T cells) from xenografted mice, revealed subsets of CD4+ effector memory (EM) and CD8+ effector (TE) T cells expressing *CCR2* and *CCR5*, consistent with NanoString data (See **Figure 4C**; **Supplementary Figure 1**). Differential gene expression analysis of CD8+ effector (TE) subset showed retention of IL-7R —albeit at low levels— indicates that IL-7R continues to support cell survival (48). Interestingly, these cells exhibited a rapid acquisition of an innate-like/effector/killing phenotype, evidenced by increased expression of cytotoxic genes, such as *PRF1*, *GZMB*, *GZMH*, and *GNLY*, alongside NK receptors including *FCGR3A* (CD16a).This suggests an integration of innate and adaptive immune responses through antibody-dependent cell-mediated cytotoxicity (ADCC) (**Figure 4D**) (49).

Furthermore, the upregulation of *NKG7* gene, which encodes a cytotoxic lymphocyte granule protein, in intratumor antigen-specific CD8+ T cells and NK cells suggests its involvement in regulating CD8+ effector T cells accumulation and activation within tumors (50). The overexpression of the *KLRK1* gene, encoding the NKG2D receptor essential for NK cell-mediated tumor killing, which engages with MIC ligands selectively upregulated on stressed or tumor cells, underscores its role in modulating T cell responses and enhancing TCR activation (51, 52).

Our analysis also revealed increased expression of the *KLRC1* gene, encoding the NKG2A receptor, which forms a heterodimeric complex with CD94 and binds to HLA-E, a non-classical MHC class I molecule. This interaction, known as “licensing,” is essential for immune regulation and tolerance. It suggests that the MHC class I environment plays a key role in calibrating the activity of CD8+ effector T cells, enhancing their ability to differentiate between tumor cells and normal cells, thereby improving their selective targeting of tumors (53, 54).

Moreover, the upregulation of *KLRG1*, an inhibitory receptor on NK cells that binds to non-MHC class I ligand cell-junction proteins, also plays a crucial role in tumor surveillance (55, 56). Similarly, the *CD300* gene, which inhibits tumor cell killing by binding to phosphatidylserine (PS), indicates another mechanism through which NY-ESO-1 T cells may fine-tune their anti-tumor responses (57). Additionally, the transcription factor *TBX21* (T-bet), acts as a pivotal regulator, driving phenotypic conversion and upregulating NK cell receptors, perforin, and granzyme, which could significantly impact the cytotoxic potential and overall functionality of CD8+ effector T cells (58, 59).

Overall, our single-cell transcriptomic analysis reveals that prolonged antigen stimulation induces notable changes in NY-ESO-1 TCR-transduced T cells, specifically within the CD8+ effector T cell subset, by 14 days post-infusion. These changes include a shift toward an NK-like CD8+ phenotype, characterized by the expression of genes associated with both NK cells and cytotoxic T lymphocytes (CTLs) (**Figure 4E**; **Table 1**) (60). This phenotypic shift indicates that NY-ESO-1 TCR-transduced CD8+ effector T cells may engage in tumor targeting through both antigen-specific mechanisms mediated by the NY-ESO-1 TCR and antigen-independent mechanisms analogous to those employed by NK cells. This dual functionality underscores the therapeutic potential of NY-ESO-1 TCR-T cells as potent tumor-eradicating agents and highlights the importance of harnessing their versatile functional capabilities to refine and enhance therapeutic strategies. Future research into the mechanisms and regulatory pathways that enhance NK-like functions in genetically modified T cells is crucial for optimizing their therapeutic efficacy.

## Data Availability

The datasets presented in this study can be found in online repositories. The names of the repository/repositories and accession number(s) can be found below: https://www.ncbi.nlm.nih.gov/geo/, GSE254553.
