# Supplementary material for: Decoding NY-ESO-1 TCR T cells: transcriptomic insights reveal dual mechanisms of tumor targeting in a melanoma murine xenograft model
Source: Front Immunol. 2024 Nov 26;15:1507218. doi: 10.3389/fimmu.2024.1507218 (PMC11628372; doi:10.3389/fimmu.2024.1507218)
Supplement: Supplementary file 1 [file DataSheet1.docx]

Supplementary Material

# Supplementary Figures and Tables

## Supplementary Figures


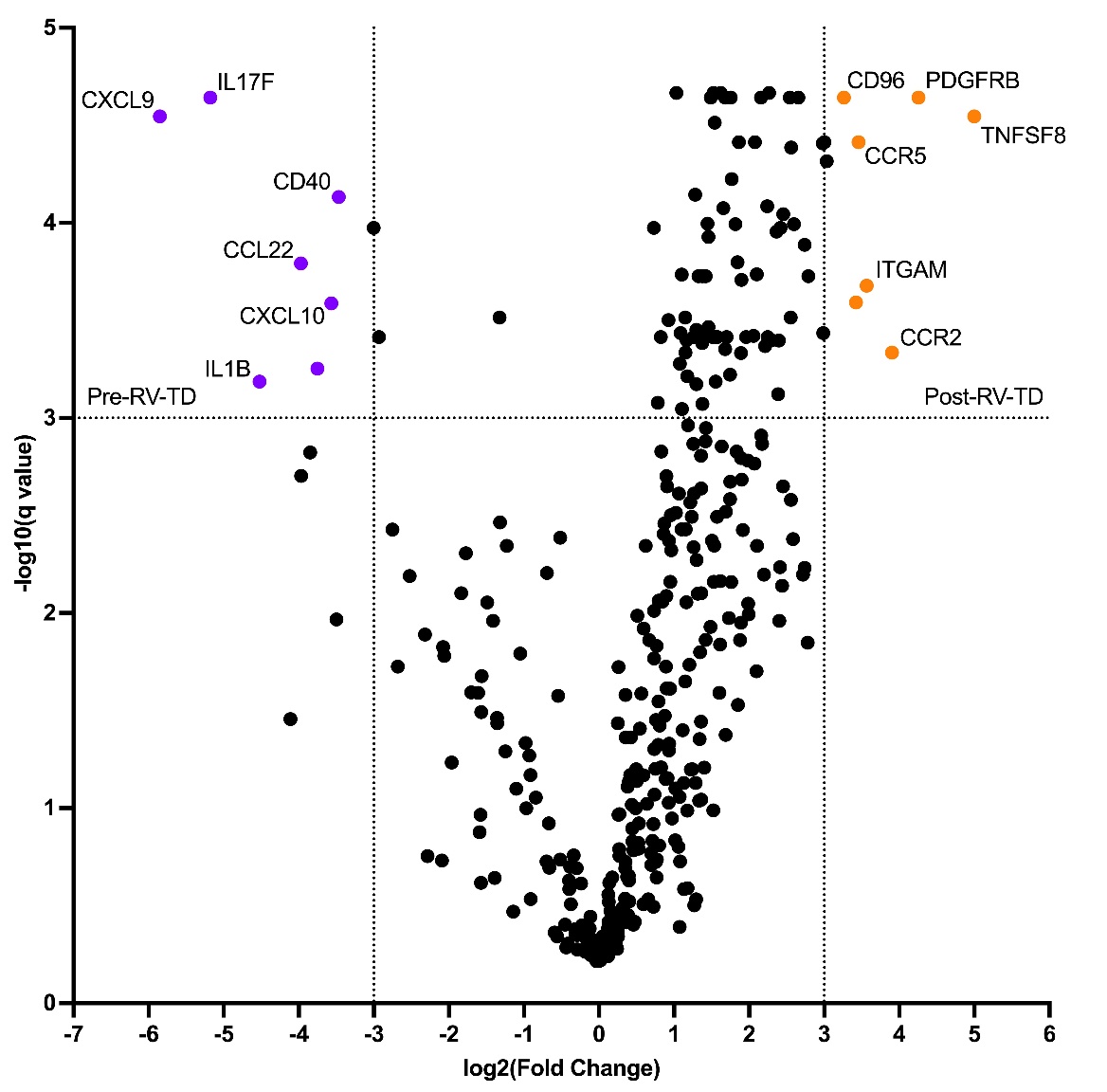


**Supplementary Figure 1.** Volcano plot of the differentially expressed genes between the NY-ESO-1-specific TCR T-cells and the non-transduced T-cells. Orange dots represent up-regulated genes and purple dots represent down-regulated genes.


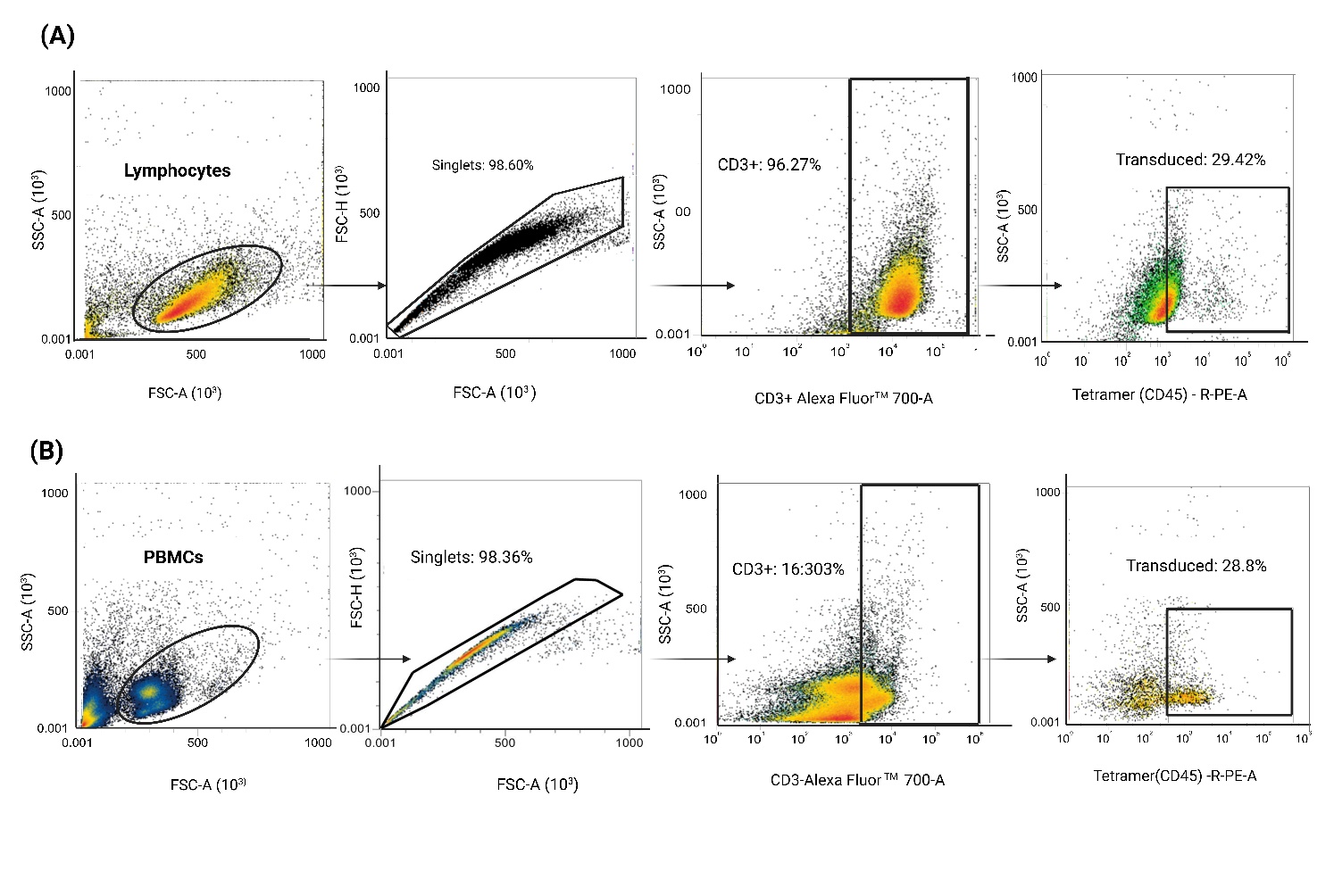


**Supplementary Figure 2.** Flow cytometric analysis of NY-ESO-1 TCR expression in infused T cells in a melanoma xenograft model. (**A**) Analysis of NY-ESO-1 TCR-transduced T cells (Control T cells) prior to infusion. (**B**) Analysis of peripheral blood T cells (PB T cells) following infusion.
